# Positive radionuclide imaging of miRNA expression using RILES and the human sodium iodide symporter as reporter gene is feasible and supports a protective role of miRNA-23a in response to muscular atrophy

**DOI:** 10.1371/journal.pone.0177492

**Published:** 2017-05-11

**Authors:** Viorel Simion, Julien Sobilo, Rudy Clemoncon, Sharuja Natkunarajah, Safia Ezzine, Florence Abdallah, Stephanie Lerondel, Chantal Pichon, Patrick Baril

**Affiliations:** 1 Centre de Biophysique Moléculaire, CNRS UPR4301, Orléans, France; 2 PHENOMIN-TAAM-UPS44, CIPA (Centre d'Imagerie du Petit Animal), CNRS Orléans, France; 3 Université d’Orléans, Collégium Sciences et Techniques, Orléans, France; Wayne State University, UNITED STATES

## Abstract

MicroRNAs (miRNAs) are key players in many biological processes and are considered as an emerging class of pharmacology drugs for diagnosis and therapy. However to fully exploit the therapeutic potential of miRNAs, it is becoming crucial to monitor their expression pattern using medical imaging modalities. Recently, we developed a method called RILES, for RNAi-Inducible Luciferase Expression System that relies on an engineered regulatable expression system to switch-ON the expression of the luciferase gene when a miRNA of interest is expressed in cells. Here we investigated whether replacing the luciferase reporter gene with the human sodium iodide symporter (hNIS) reporter gene will be also suited to monitor the expression of miRNAs in a clinical setting context. We provide evidence that radionuclide imaging of miRNA expression using hNIS is feasible although it is not as robust as when the luciferase reporter gene is used. However, under appropriate conditions, we monitored the expression of several miRNAs in cells, in the liver and in the tibialis anterior muscle of mice undergoing muscular atrophy. We demonstrated that radiotracer accumulation in transfected cells correlated with the induction of hNIS and with the expression of miRNAs detected by real time PCR. We established the kinetic of miRNA-23a expression in mice and demonstrated that this miRNA follows a biphasic expression pattern characterized by a loss of expression at a late time point of muscular atrophy. At autopsy, we found an opposite expression pattern between miRNA-23a and one of the main transcriptional target of this miRNA, APAF-1, and as downstream target, Caspase 9. Our results report the first positive monitoring of endogenously expressed miRNAs in a nuclear medicine imaging context and support the development of additional work to establish the potential therapeutic value of miRNA-23 to prevent the damaging effects of muscular atrophy.

## Introduction

MicroRNAs (miRNAs) are a class of small non-coding RNAs that regulate gene expression at the post-transcriptional level by binding mainly to the 3'-end of mRNA transcripts to induce translational repression and/or mRNA degradation [1]. This novel mode of post-transcriptional gene regulation has attracted considerable attention from all areas of biology because of its remarkable conservation between species and its crucial role in many key biological events from cell fate in development to cell differentiation, proliferation and apoptosis. Today more than 2 000 mature miRNAs have been annotated in the human genome and are predicted to control at least 60% of protein-coding genes. Therefore, it is not surprising that miRNA deregulation is a hallmark of many diseases and that modulation of miRNA function using synthetic miRNA antagonists or agonists could reverse the pathological state of diseases as demonstrated in many preclinical animal models and also in humans [2, 3].

To accelerate the translation of miRNAs to the clinic, imaging probes compatible with clinical practice are required. This task is challenging mainly because of (i) the tiny size of miRNAs, which limits the development of a broad range of miRNA imaging probes; (ii) the dynamic expression pattern of miRNAs that make difficult the capture a miRNA expression pattern at a specific time point; (iii) the difficulty of monitoring the functional active form of miRNAs processed by the miRISC machinery; and (iv) the lack of imaging modalities compatible for human use. Nevertheless, some miRNA imaging probes have been successfully developed in preclinical animal models. They can be subtyped in two categories: probes using reporter genes (biological probes) and probes using fluorescent oligonucleotides (synthetic probes) [4, 5]. Even though fluorescent synthetic probes have shown promising results in cells and in small animals, they cannot yet be translated to the clinic because of their low signal-to-background ratios and poor tissue penetration of light excitation and emission. In contrast, molecular imaging probes using reporter genes are more sensitive because even when transfected in cells with low efficacy, the amount of reporter protein produced by the cellular machinery is always greater than what can be achieved upon delivery of synthetic exogenous probes. Some reporter genes have a long clinical history in nuclear medicine. This is the case for the herpes simplex virus type 1 thymidine kinase (HSV-tk), the dopamine D_2_ receptor (D_2_R), the somatostatin receptor subtype 2 (SSTR2) and the sodium/iodide symporter (NIS) [6]. NIS has been used for over 70 years to diagnose and treat human thyroid diseases [7]. It is a 13 membrane spanning glycoprotein expressed on the basolateral surface of thyroid follicular cells where it is responsible for the active transport of iodide from blood to the thyroid gland for the synthesis of T3 and T4 hormones. This unique biological property has been exploited to accumulate radioiodine isotopes (^123^I^-^, ^124^I^-^, ^125^I^-^ and ^131^I^-^) and other radiopharmaceuticals (^99m^TcO_4_^-^, ^211^As, ^186,188^Re) in the thyroid of patients to image the size, shape and position of thyroids using PET, SPECT and scintigraphy [7]. In addition to its diagnostic potential, NIS also has a therapeutic potential. When radiopharmaceuticals with high energy deposit (^186,188^Re, ^211^As, ^131^I^-^) are used, the decay of radioisotopes leads to the emission of alpha and/or beta particles and gamma rays that are toxic for the cells, a well-known process referred to as internal radiation therapy. This dual property has also attracted significant interest in the field of gene therapy. In the last 15 years, several groups have used gene therapy approaches to transfer the hNIS gene into non-thyroidal cancer cells to treat these cancer types as efficiently as thyroid cancer. It was demonstrated that the human NIS (hNIS) is a relevant nuclear medicine reporter gene for monitoring the kinetic and location of hNIS gene transfer in animal models, providing biosafety information before the administration of therapeutic doses of radioisotopes [8, 9]. Recently, the clinical imaging potential of hNIS was validated in cancer patients administrated with oncolytic virus encoding hNIS to monitor the spread of the virus at the whole body scan of the patients [10–13]. It was concluded that this imaging modality is possible and safe in humans and that the images collected provide an opportunity to predict treatment outcome and responsiveness and also to rationalize the design of novel generations of more potent oncolytic viruses for human use [14].

In the miRNA field, two recent independent studies have shown that the monitoring of miRNA expression is possible using hNIS as reporter gene and a gamma camera. This approach was used to visualize the expression of miRNA-9 [15] and -16 [16] in neural and gastric cancer cells upon engraftment in nude mice. The miRNA monitoring probe used is based on the well-established miRNA-OFF system [5, 17] in which a complementary block sequence specific to a miRNA is subcloned in the 3’-UTR part of the hNIS gene. Therefore, when functional in cells, miRNA represses the expression of hNIS in cells, resulting in the reduction of radioiodine uptake and a decrease of radioisotope signals in mice. Although relevant, this methodology suffers from some drawbacks associated with the fact that the detection of miRNA expression is signed by a loss of imaging signals. Recently, we developed a miRNA-ON system, called RILES, standing for RNAi-inducible expression system, that relies on an engineered genetic switch expression system to switch-ON the expression of the luciferase gene when a miRNA of interest is present in cells [18]. Therefore when miRNA is expressed in cells, its expression is signed by the emission of bioluminescence signals that can be monitored using standard bioluminescence equipment.

Here we examined whether it might be possible to monitor the endogenous expression pattern of miRNA in a clinical setting using hNIS as reporter gene, ^99m^TcO_4_^-^ as radiotracer and a SPECT/CT camera. We replaced the luciferase reporter gene by the hNIS reporter gene and generated a RINES system, standing for RNAi-inducible hNIS reporter expression system. We evaluated the feasibility of this approach by monitoring changes in radiotracer uptake in cells transfected with synthetic miRNA *in vitro*, the expression of miRNA 122 in the liver of live-anesthetized mice and the expression of miRNA candidates in response to muscular atrophy, used as an experimental animal model of pathology. We established, in real time, the kinetic of miRNA-23a expression during development of this disease and examined the potential implication of the miRNA 23a/APAF-1/Caspase 9 axis of regulation in the apoptosis of skeletal muscle undergoing muscular atrophy.

## Materials and methods

### Tissue culture, reagents

Hela cells were obtained from ATCC, cultured in 4.5 g/l Dulbecco’s modified Eagle’s medium supplemented with 10% (v/v) of fetal bovine serum and with penicillin and streptomycin and maintained in a humidified atmosphere of 5% CO_2_ at 37°C. Cumate (4-isopropylbenzoic acid), sodium perchlorate (NaCIO_4_) and sodium iodide (NaI) were purchased from Sigma-Aldrich (Sigma-Aldrich, USA). The Lipofectamine RNAiMAX transfection reagent, synthetic microRNA mimics and RIPA lysing buffer were from Thermofisher (Thermofisher, USA). The Pluronic F-68 formulation used to transfect the tibialis anterior muscle *in vivo* was also purchased from Sigma Aldrich. The human specific NIS antibody (clone 17795) was from Abcam (Abcam, USA), the APAF-1 (clone D5C3), Caspase 9 (clone 353) antibodies were from Cell signalling Technologies (Cell signalling Technologies, USA) and the tubulin (clone TU-02) antibody was from Santa Cruz Biotechnology. The luciferin substrate for *in vivo* use was purchased from Promega (Promega, USA). The ^99m^TcO_4_ pertechnetate (^99m^TcO_4_^-^) was from IBA Molecular (900 MBq, IBA Molecular, France) and was collected freshly from the Nuclear medicine department of a neighboring hospital (Centre Hospitalier Regional d’Orléans, Orléans, France) and used within the day.

### RINES plasmids construction

The hNIS cDNA (SLC5A5, NM_000453) was amplified by PCR from the pCMV6-Entry plasmid purchased from OriGene Technologies (OriGene Technologies, USA). The RINES plasmids were constructed by replacing the firefly luciferase cDNA from the RILES plasmids [18] with the hNIS cDNA generated by PCR. The hNIS PCR fragments were flanked by Pme I and BamH I restriction enzyme sequences to facilitate the subcloning in the RILES plasmids digested with the same restriction enzymes. The miRNA targeting sequences (miR T) used to place the RINES plasmids under control of miRNA-122, -206, -486 and -23a were prepared as previously described and are listed in the S1 Table. The miR T sequence containing 4 complementary bound sequences of each miRNA was designed from the miR base 21 data base. The sequence and the functionality of all generated plasmids were sequenced-verified and tested with Cumate, used as a gene-switch agent. Plasmids were amplified using Endofree plasmid kits (Qiagen, USA).

### Transfection, luciferase activity and ^99m^TcO_4_^-^ uptake quantification *in vitro*

Hela cells (1 x 10^5^) were plated in triplicate in 24-wells plates and transfected the following day with plasmids and miRNA mimics according to manufacturer instructions (RNAiMax, Thermofisher). When indicated, the Cumate was added to the cell supernatants 4 hours after transfection at indicated concentrations. The relative luciferase units were determined 48 hours after transfection of cells with the pRILES plasmids and normalized to protein content using the BCA assay (Thermofisher) as previously described [18]. The relative ^99m^TcO_4_^-^ uptake values in the pRINES-transfected cells were also determined 48 hours post transfection following a well-established protocol [19, 20]. In brief, cell monolayers were incubated for 45 min at 37°C, 5% CO_2_ with Hank’s balanced salt solution (HBSS) supplemented with 10 μmol/L of NaI and 1μCi (37 kBq) of ^99m^TcO_4_^-^ with or without addition of sodium perchlorate (100 μM). Then the cell monolayers were quickly washed once with 2 ml of ice-cold HBSS and immediately lysed with 300 μl of RIPA lysing buffer. After incubation of 5 min on ice, the cell monolayers were scratched mechanically and radioactivity measured using a gamma counter designed from a small animal gamma Imager (Biospace Lab, France) whose parallel collimator was replaced by a circular low noise shielding. Quantification of radioactivity counts in lysates was determined using the winTMCA 1.617 software (FLIR Systems, France).

### Quantitative real time PCR analysis

Total RNA extraction was performed as previously described [18]. Tibialis anterior skeletal muscle tissues were lysed in 1/20 (w/vol) of lysis binding buffer (mirVana microRNA isolation kit, Ambion, US) followed by tissue homogenization using a Precelyss 24 Unit (Precelyss, Bertin, France) and CKMix ceramic beads (Ozyme, Paris, France). The short and long RNAs were collected simultaneously using the miRNAVANA isolation kit. RNA integrity was assessed using a BioAnalyzer 2100 (Agilent technologies). Samples with an RNA integrity number (RIN) superior or equal to 8 were considered for further analysis. The NCode VILO miRNA cDNA synthesis kit was used to generate cDNAs for both mRNA and miRNA analysis. Real time quantitative PCR was performed with Sybergreen dyes (QuantiFast SYBR Green master mix, Qiagen) using specific primers listed in S1 Table. The specificity of the PCR amplicon (size and product) and absence of primer-dimer were verified by melt-curve analysis using LightCycler 480 equipment and software (Roche). Samples were normalized to the 6S rRNA level for quantification of the mRNA transcript and with the snU6 level for quantification of mature miRNA. The relative expression levels of miRNAs and mRNAs were determined using the standard 2^-ΔΔ^CT Livak and Schmittgen method.

### Confocal microscopy analysis

HeLa cells were seeded at 1 x 10^5^ cells per well in 24-well plates containing glass cover slips. On the following day, cells were transfected with the pRINES/122T plasmid and incubated with Cumate for 48 hours. Cells were fixed and permeabilized with cold methanol for 20 min, washed in PBS, incubated with the hNIS antibody (1/100 dilution) for 40 min at room temperature followed by an Alexa Fluor 488 goat anti-mouse IgG (H + L) secondary antibody (Invitrogen) for 30 min at room temperature and washed again with PBS. Then, the cover slips were mounted on slides using Vectashield mounting medium (Vector Laboratories, USA) for confocal microscopy observation [21]. Slides were analysed using an LSM 510 META scanning device coupled to an Axiovert 200 microscope (Carl Zeiss, Germany), using the 63 x high numerical-aperture oil immersion objective lenses. Image sizes were set to 512 × 512 pixels. Z-stack images were acquired randomly for each condition, and representative images from the focal plane of the nucleus were analysed by Image J v1.4.9 software. Regions of Interest (ROIs) were selected using the polygon select tool of the software and the mean fluorescence intensity (MFI) determined. For quantification studies, ratios between MFI expressed in pixels and the selected area of ROIs expressed as relative unit of surface (RUS) were calculated and plotted on graphs.

### Immunohistochemistry procedure

Tissues were collected at autopsy of mice and immediately fixed in 3% paraformaldehyde, paraffin embedded, and cut into 6-mm-thick sections. Immunostaining of hNIS was performed as previously described [22] using the hNIS specific antibody (1/100 dilution) incubated overnight at 4°C. The tissue sections were then counterstained with routine Harris' Hematoxylin staining procedure (Sigma Aldrich, USA).

### Animal experiments

Animal housing was carried out in our animal facility (accreditation number D-45-234-12, Chantal Pichon) according to the guidelines of the French Ministry of Agriculture for experiments with laboratory animals (Law 87848 Experimental procedures were approved by a local ethical committee (Comité d'Ethique pour l'Expérimentation Animale, Campus d'Orléans, France, French Registration CECCO 03) according to the document file n°1043 provided. Six- to eight-week-old female BALB/c mice (Charles River Laboratoire, France) were allowed to acclimatize for 1 week and kept in individual ventilated cages with free access to food and water. Procedures for intramuscular and hydrodynamic administrations were performed as previously described [18], except that the amount of RINES plasmids administered was 5 μg for the hydrodynamic injections and 2 μg for the intramuscular injections. For the latter, plasmids were formulated with the Pluronic F-68 formulation (Sigma Aldrich) as detailed in [23]. Procedures to induce muscular denervation of one of the lower legs of the animals were exactly the same as those previously described [18, 23]. At the end of experiments, mice were anesthetized with isoflurane and sacrificed by cervical dislocation.

### Nano-SPECT/Computer tomography imaging

Small-animal SPECT/CT (Single Photon Emission Computed Tomography) scanning was performed as previously described [24]. At indicated times after administration of RINES plasmids, animals were anesthetized and then given an intravenous injection of ^99m^TcO_4_^-^ (18.5 MBq). Mice were then positioned inside a SPECT/CT scanner (Mediso Medical Imaging system, Hungary) equipped with a 0.8 mm multi-pinhole resolution. A first computed tomography (CT) scan was acquired using the Nucline software (v3.02, Mediso Medical Imaging system) to obtain an anatomical reference frame. Then SPECT images were acquired exactly 30 minutes after injection of ^99m^TcO_4_^-^. Acquisition time depended on the specific radioactivity levels in each mouse so as to collect at least 40 000 counts per projection. Reconstruction of SPECT images was performed with the HiSPECT (v1.4.24.21, Scivis) software. The fusion of SPECT and CT images was obtained using the VivoQuant (v1.23, InVicro) software. From the reconstructed images, ROIs were drawn automatically except for liver and stomach in which ROIs were drawn manually using the VivoQuant (v1.23, InVicro) software. Radioactivity counts within ROIs were then quantified using the VivoQuant software.

### *In vivo* bioluminescence imaging

Bioluminescence imaging was performed as previously described [18, 23]. Briefly, mice received intraperitoneally *in vivo* 2 mg of Luciferin substrate and were isoflurane-anesthetized 5 min later. Then, after a further 5 min (10 minutes after injection of the luciferin substrate), the mice were placed in an IVIS Lumina II imaging scanner (PerkinElmer, USA) coupled to an isoflurane ventilation system to maintain constant anaesthesia. The acquisition parameters were 2 min integration time, F/Stop = 1, binning 8. Bioluminescence signals were collected from ROIs drawn manually and quantified using the living Image Software (Perkin Elmer). The final data were expressed as photons/second/pixel/sr. The sensitivity of the imaging scanner was tested monthly with commercially available positive sources of bioluminescence.

### Western-blot analysis of tissue samples

Tibialis anterior skeletal muscle tissues collected at autopsy of mice were lysed in RIPA buffer supplemented with freshly prepared protease cocktail inhibitors (Sigma Aldrich) using the tissue homogenizer Precelyss 24 Unit (Bertin Instruments, France) and CKMix ceramic beads (Bertin Instruments). Lysates were clarified by centrifugation at 10 000 g for 5 min before quantifying protein content using the BCA assay. Then 50 μg of tissue lysate proteins were resolved on 8% or 10% SDS-PAGE and transferred to nitrocellulose membranes. After a blocking step, membranes were sequentially incubated with the primary antibodies and then with secondary antibodies conjugated to horseradish peroxidase. Reactions were visualized by chemiluminescence reactions (Clarity Western-blot ECL substrate, BioRad, USA) with autoradiography films. Quantification of protein bands was performed by using the ImageJ v1.4.9 software.

### Statistical analysis

The experiments were done at least three times in triplicate. Error bars represent SD or SEM. Data showing comparisons between two groups were assessed using Student’s t-test. P values ≤ 0.05 were considered statistically significant.

## Results

### Molecular construct, design and functionality

**Fig 1:**
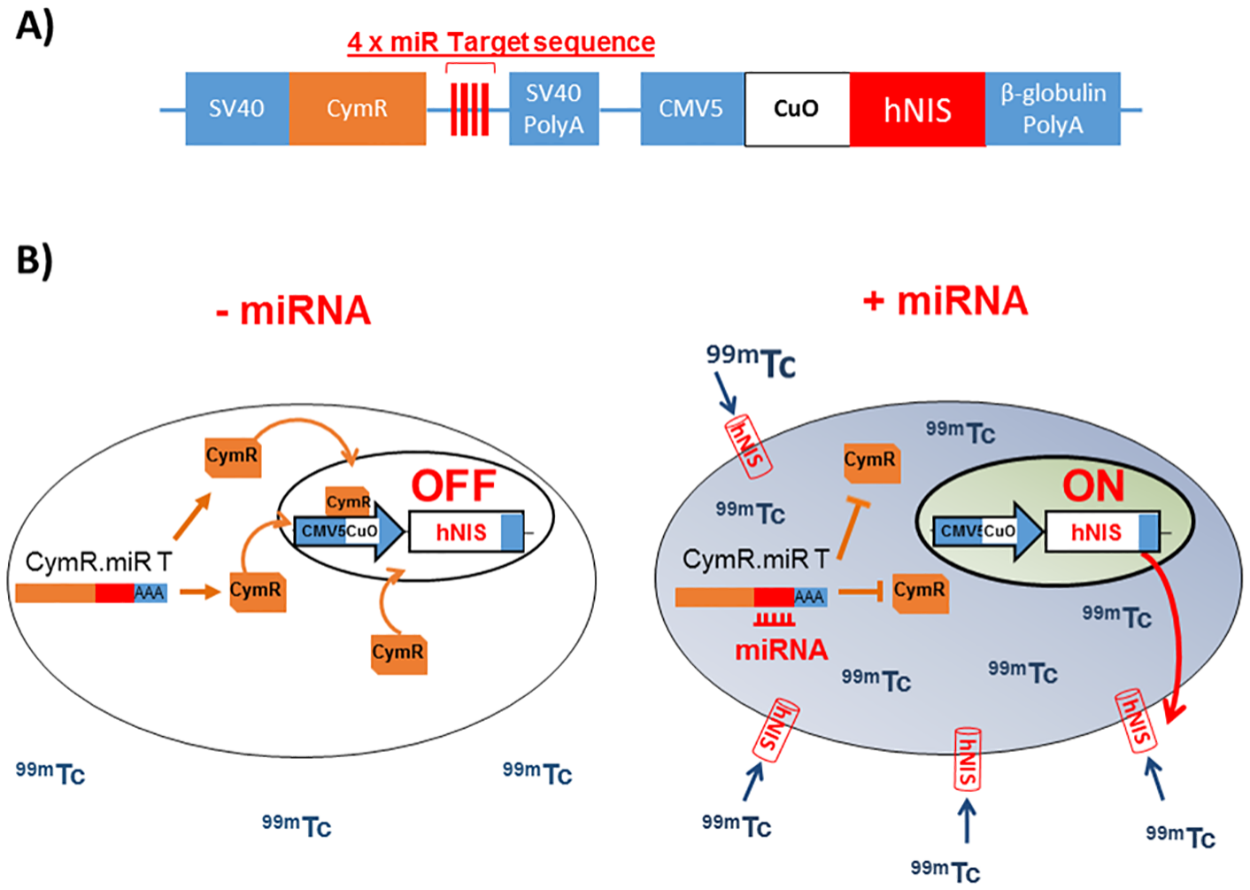
Schematic illustration of the principle of the RINES system. A) Representation of the RINES plasmid construct with localization of the miRNA targeting sequence (miR T) and the hNIS cDNA reporter gene. B) Left panel, when the miRNA of interest is not expressed in cells, the CymR mRNA is transcribed as well as the CymR protein which binds to the Cuo operator sequence located in the CMV5/Cuo inducible expression cassette. Under this configuration, hNIS expression is switched-OFF and there is no, or low ^99m^Tc0_4_^-^ accumulation in cells. Right panel, in contrast when present in cells, the miRNA of interest binds to the miR T sequence located in the 3′-UTR of the CymR repressor transcript, inhibiting production of the CymR protein through activation of the RISC machinery. Under this configuration, hNIS is switched-ON, resulting in expression of hNIS at the cellular membrane and accumulation of ^99m^Tc0_4_^-^ in cells.

We first subcloned the hNIS reporter gene in the RILES plasmid to generate a novel system called RINES, for RNAi-inducible hNIS expression system. As described in Fig 1, the RINES system was designed in such a way that the presence of the miRNA of interest is able to induce hNIS expression, providing a positive molecular miRNA imaging system. To generate this ON signal, we subcloned, in the 3′- UTR of the CymR transcriptional repressor cDNA, four complementary block sequences of a given miRNA. Therefore when expressed in cells, the miRNA binds to CymR transcriptional repressor mRNA through a perfect base pairing mechanism inducing its degradation through activation of the RISC machinery. In the absence of transcriptional repressor bound to the Cuo operator, located in the second expression cassette, the hNIS reporter gene is transcribed and the hNIS protein produced. The addition of a gamma emitting radiotracer such as ^99m^TcO_4_^-^ results in the intracellular accumulation of radiotracers in the RINES expressing cells that can be monitored using a 2D planar gamma counter for *in vitro* studies and a SPECT camera for *in vivo* analysis. In this study, we denoted the RINES plasmids as follows: pRINES/122T when the RINES plasmid contains 4 complementary block sequences to the miRNA-122, and pRINES/23aT when the RINES plasmid contains 4 complementary block sequences to the miRNA-23a. As control, we used the RINES plasmid (pRINES) that did not contain any miRNA targeting sequence. The complete list of RINES plasmids constructed is shown in S1 Table.

We first performed a functional validation study using Cumate as switching-ON agent. As for other genetic-switch expression systems [25], the Cym operon can also be switched-ON using small exogenous molecules, in this case Cumate [26] that, once bound to the CymR transcriptional repressor protein, changes its conformation impeding its binding to the Cuo operator. As a result, the hNIS expression cassette is switched-ON, resulting in ^99m^TcO_4_^-^ uptake in cells.

**Fig 2:**
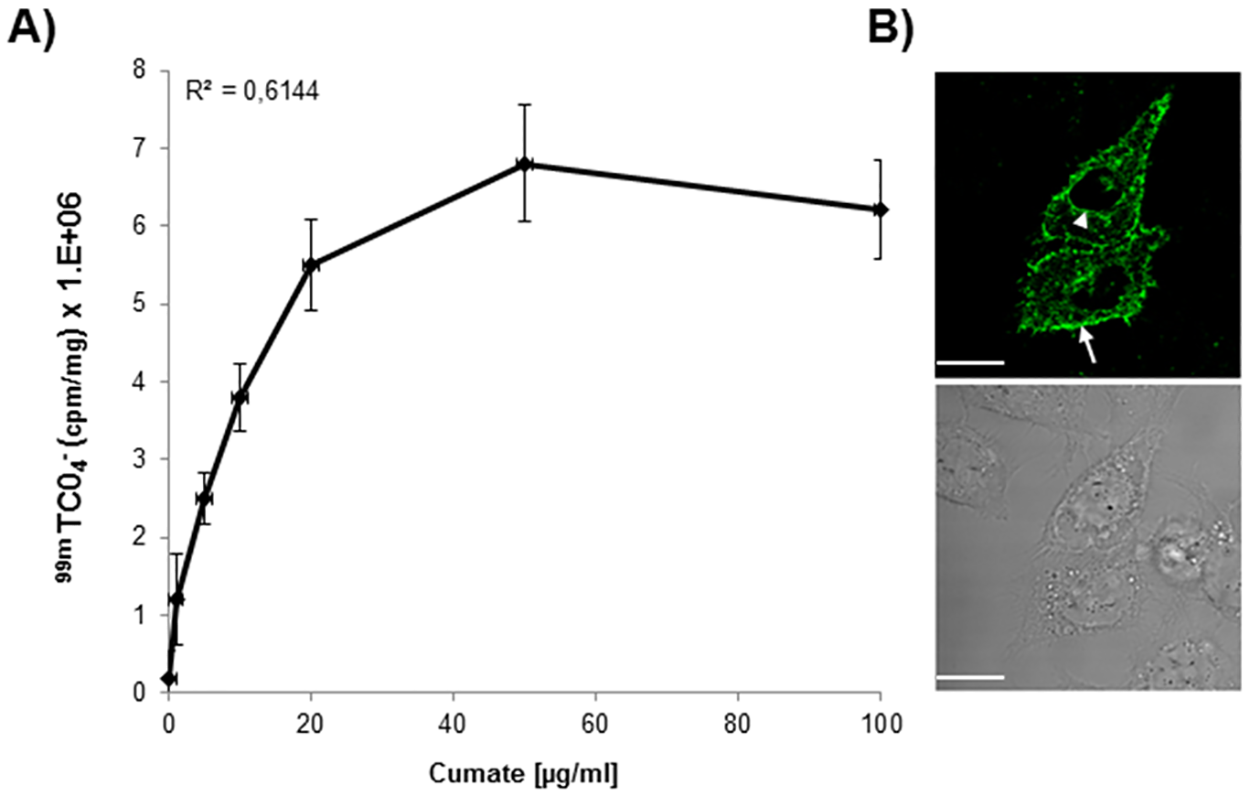
Functional validation of the RINES system using Cumate as switching-ON inducer. (A) ^99m^Tc0_4_^-^ uptake in cells transfected with the pRINES/122T and treated with increasing concentrations of Cumate to switch-ON the expression of hNIS. Inset is the correlation coefficient value. Data shown are the mean ±SD of one representative experiment performed in triplicate and reproduced at least three times. B) Confocal immunofluorescence analysis of hNIS location in Hela cells transfected with the pRINES/122T and treated with Cumate (100 μg/ml). Upper panel is a representative picture of immunofluorescence staining obtained with a specific hNIS antibody. Arrow and arrowhead indicate the location of hNIS protein at the plasma membrane and in the perinuclear region of transfected cells respectively. Lower panel is a representative bright field picture of the same cells as in the upper panel. Scale, 10 μM.

As shown in Fig 2A, addition of ^99m^TcO_4_^-^ 48 hours after transfection of cells with the RINES/122T plasmid and treatment with an increased concentration of Cumate, resulted in a gradual increase in ^99m^TcO_4_^-^ uptake values, up to the concentration of 50 μg/ml of Cumate. Above this concentration, ^99m^TcO_4_^-^uptake in cells reached a plateau and thus saturated. No significant differences (P = 0.82) were detected between the means of radioactivity detected in cells treated with 50 and 100 μg/ml of Cumate (6.8 ± 0.78 x 10^6^ and 6.2 ± 0.63 x 10^6^ respectively). As a result, ^99m^TcO_4_^-^ uptake in pRINES/122T transfected cells did not correlate well (R^2^ = 0.61) with the concentration of Cumate used. To confirm that ^99m^TcO_4_^-^ uptake in cells correlated with the ectopic production of hNIS protein, we performed an immunofluorescence staining analysis using a commercially available hNIS specific antibody. As expected, hNIS protein expression was detected at the cell membrane of the pRINES/122T-transfected cells although a significant fraction of hNIS staining was also detected in the perinuclear regions of cells (Fig 2B).

### Dual comparison between the RILES and the RINES systems

The saturable uptake of ^99m^TcO_4_^-^ in transfected cells at high Cumate concentration was unexpected. Such a saturation of the read-out signals in the pRILES transfected cells was not observed in our previous study [18]. To investigate this point, we made a dual comparison between the RILES system that employed the luciferase gene as reporter gene and the RINES system that employed hNIS as reporter gene.

**Fig 3:**
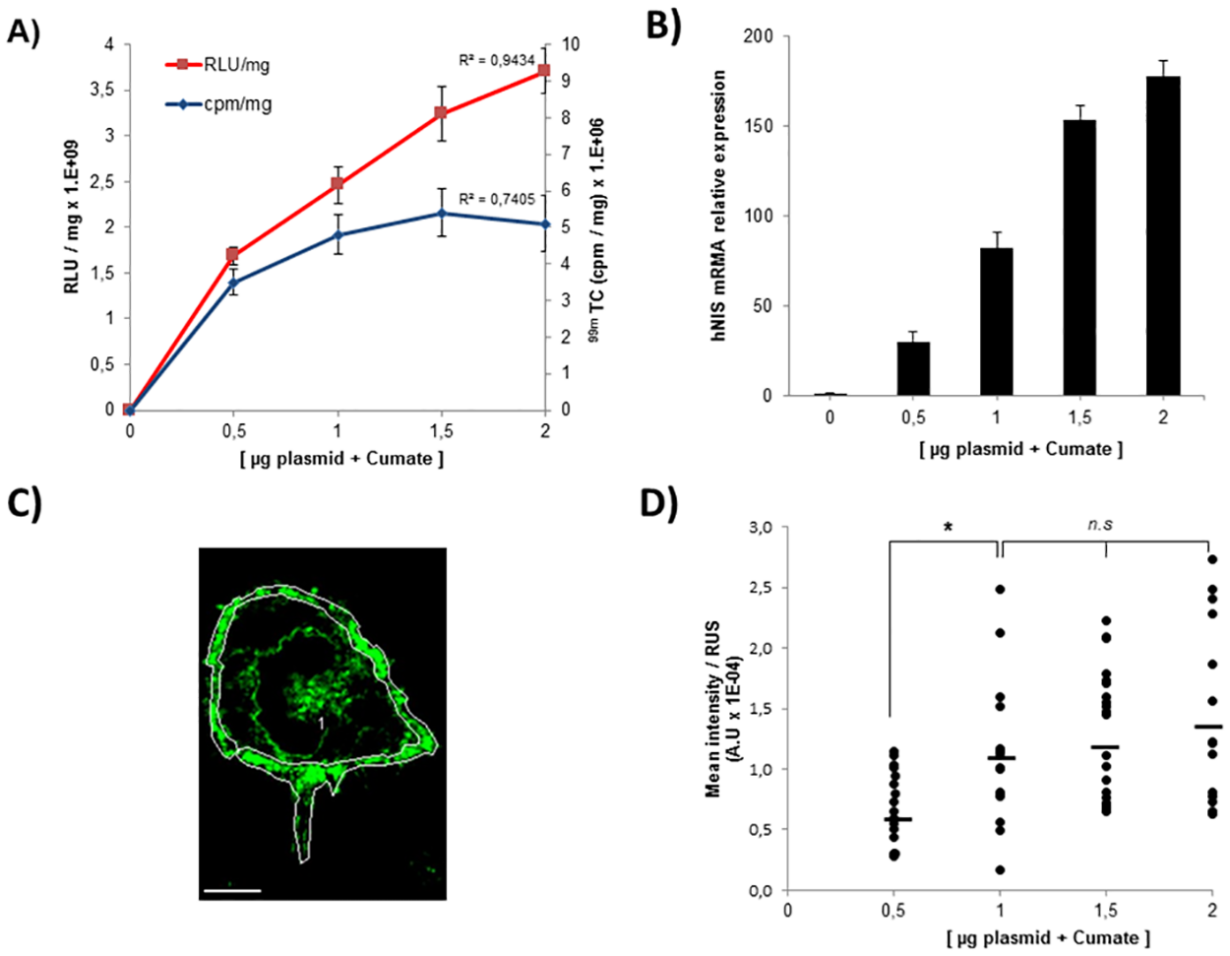
Expression of hNIS at the cell membrane is saturable and leads to saturation of ^99m^Tc0_4_^-^ uptake in RINES transfected cells. A) Dual comparison of the read-out performance of the RILES system that employed the luciferase reporter gene (red line) and the RINES system that employed the hNIS reporter gene (blue line). Inset are the correlation coefficient values determined from the two different curves. B) Quantitative RT-PCR analysis of hNIS transcripts produced in Hela cells upon transfection of pRINES/122T plasmids and treatment with Cumate. C) is a representative example of pictures collected by confocal immunofluorescence analysis performed with an hNIS antibody and processed with the image J software to draw the region of interest (ROI, white line) covering the cell periphery of the pRINES/122T-transfected cells treated with Cumate. Scale, 5 μM. D) Quantitative analysis of C performed from a pool of randomly selected transfected cells for each experimental condition indicated. Horizontal bars represent the mean values of fluorescence intensity calculated from D. Data shown in Fig 3A and B are the mean ±SD of one representative experiment performed in triplicate and reproduced at least three times. Statistical analysis in Fig 3D was performed by the two-tailed t -test, * P < 0.05; n.s (no statistically significant difference).

Hela cells were transfected with increasing amounts of pRILES/122T or pRINES/122T plasmids and incubated with 100 μg/ml of Cumate to switch-ON the expression of reporter genes in cells. The final read-out was measured using either a luminometer to quantify photons emitted from the luciferase reaction or a gamma counter to quantify the accumulation of ^99m^TcO_4_^-^ in cells expressing hNIS. The raw data were normalized to protein content and plotted on the same graph according to plasmid concentration. As shown in Fig 3A, the relative luciferase unit values per milligram of protein (RLU/mg) detected in pRILES/122T transfected cells correlated well (R^2^ = 0.94) with the amount of pRILES/122T plasmids transfected in cells and incubated with Cumate. In contrast, the relative ^99m^TcO_4_^-^ uptake values per milligram of protein (cpm/mg) detected in the pRINES/122T transfected cells correlated much less (R^2^ = 0.74). No significant difference (p = 0.88) was detected between the mean of radioactivity detected in Hela cells transfected with 1.5 μg and 2 μg of pRINES/122T (5.4 ± 0.32 x 10^6^ versus 5.1 ± 0.25 x 10^6^).

To investigate the mechanism responsible for the saturation of ^99m^TcO_4_^-^ uptake in these cells, we performed a similar experiment and quantified the production of hNIS mRNA by quantitative RT-PCR. Results in Fig 3B show that by contrast to what was found in Fig 3A, a dose-response relationship was detected between the hNIS mRNA and the amount of plasmids used to transfect the cells. This result indicated that the saturation of radiotracer uptake in pRINES/122T-transfected cells could not be explained by a saturation of hNIS mRNA production. Therefore, we quantified the expression of hNIS protein located at the plasma membrane of the cells. We performed this analysis by confocal immunofluorescence microscopy to distinguish the location of hNIS at the plasma membrane from hNIS in the perinuclear region of the cells. The Image J software was used to quantify the relative immunofluorescence intensity of the region of interest (ROI) covering the whole cell periphery of transfected cells. An illustrative example of the procedure used is shown in Fig 3C. Results of this analysis (Fig 3D) indicated that the expression of hNIS at the plasma membrane was heterogeneous in all experimental conditions tested and tended to increase when the amount of pRINES/122T used to transfect the cells shifted from 0.5 to 1 μg. Indeed significant difference (P = 0.012) was detected when 0.5 μg (0.68 ± 0.29 x 10^−4^) and 1 μg (1.12 ± 0.57 x 10^−4^) of pRINES/122T was used to transfect the cells. In contrast, when a higher amount of pRINES/122T plasmid was used, the expression of hNIS at the cell membrane saturated and again reached a plateau. Indeed, no statistically significant difference was found between the means of fluorescence detected in Hela cells transfected with 1 (1.12 ± 0.57 x 10^−4^), 1.5 (1.27 ± 0.51 x 10^−4^) and 2 μg (1.39 ± 0.71 x 10^−4^) of pRILES/122T. These results indicate that the saturation of ^99m^TcO_4_^-^ uptake in pRINES/122T cells could be explained by defective hNIS protein processing to the plasma membrane when a high amount of plasmid is used.

### Functional monitoring of exogenously expressed miRNA mimics

We then examined whether synthetic miRNA mimics delivered in cells might also be used as inducers to switch-ON the expression of hNIS in the pRINES transfected cells and if ^99m^TcO_4_^-^ uptake in cells reflected the concentration of these miRNAs.

**Fig 4:**
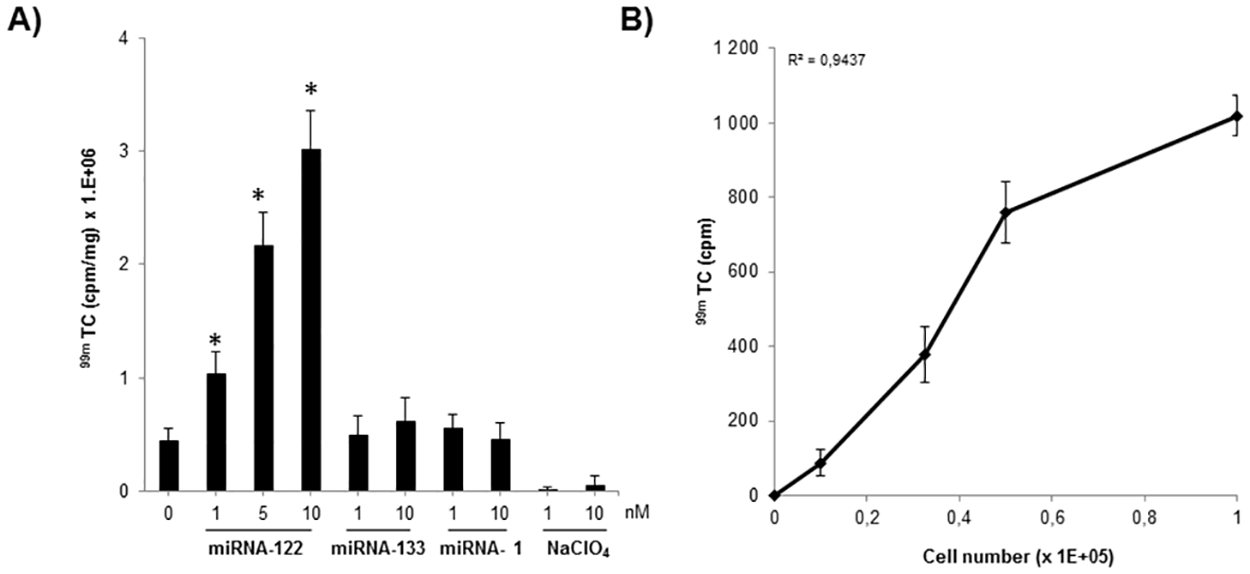
^99m^Tc0_4_^-^ uptake in Hela cells transfected with the pRINES/122T plasmid and miRNA mimics. A) Hela cells were transfected with pRINES/122T in presence of indicated concentrations of miRNA-122 mimic or, as a control, miRNA-133 and -1 mimics. For a set of experiments, sodium perchlorate (NaClO_4_), an inhibitor of hNIS-mediated ^99m^Tc0_4_^-^ uptake, was added to the cells to assess specificity of the results. (B) ^99m^Tc0_4_^-^ uptake in Hela cells correlated with the number of Hela cells transfected with the pRINES/122T and treated with Cumate (100 μg/ml). Inset is the correlation coefficient value. Data shown are the mean ±SD of one representative experiment performed in triplicate and reproduced at least three times. Statistical analysis in Fig 4A was performed by a two-tailed t-test, * P ≤ 0.05 compared to control cells, not transfected with miRNAs.

In a pilot experimental study, we found that transfection of 0.5 μg of RINES/122T plasmid in cells provided the best linear read-out to detect miRNA mimics transfected in cells. We therefore selected this amount of pRINES/122T to transfect the Hela cells in presence of increasing concentrations of miRNA-122 mimic. As shown in Fig 4A, increasing concentrations of synthetic miRNA-122, from 1 to 10 μM, also increased ^99m^TcO_4_^-^ uptake values detected in these cells. Indeed, the means of radioactivity were 1.36 ± 0.25 x 10^6^ cpm/mg, 2.76 ± 0.41 x 10^6^ cpm/mg and 3.41 ± 0.52 x 10^6^ cpm/mg, when 1 nM, 5 nM and 10 nM of miRNA-122 mimic were delivered to the cells respectively. Noteworthy, all of these mean values were found to be significantly different (P ≤ 0.05) from the mean value detected in control cells, not transfected with miRNA mimics (0.45 ± 0.041 x 10^6^). We conducted a similar set of experiments with control mimics (data not shown) or an irrelevant miRNA mimic (miRNA-133 or -1). The mean values of ^99m^TcO_4_^-^ uptake detected in these cells were similar (P ≥ 0.05) to that detected in control cells, not transfected with miRNA mimics. Furthermore, the addition of sodium perchlorate, a well-known specific inhibitor of hNIS-mediated ^999m^TcO_4_^-^ uptake totally inhibited accumulation of radiotracer in the pRINES/122T-transfected cells. We finally examined whether ^99m^TcO_4_^-^ uptake in cells might also correlate with the number of pRINES/122T transfected cells. We transfected a bulk of Hela cells with the pRINES/122T and loaded serial dilution of these cells in 24-wells plates before treating the cells with Cumate to switch-ON hNIS expression. Then 24 hours later, ^99m^TcO_4_^-^ uptake in these cells was quantified and plotted as function of cell density. As shown in Fig 4B, there is a clear dose-response correlation (R^2^ = 0.94) between the means of radiotracer uptake values detected and cell numbers.

### Real time monitoring of endogenously expressed miRNA in the liver of the mice

Next, we evaluated whether it might be possible to monitor in real-time the expression of endogenously expressed miRNAs in live-anesthetized mice using a SPECT/CT camera and ^99m^TcO_4_^-^ as radiotracer.

**Fig 5:**
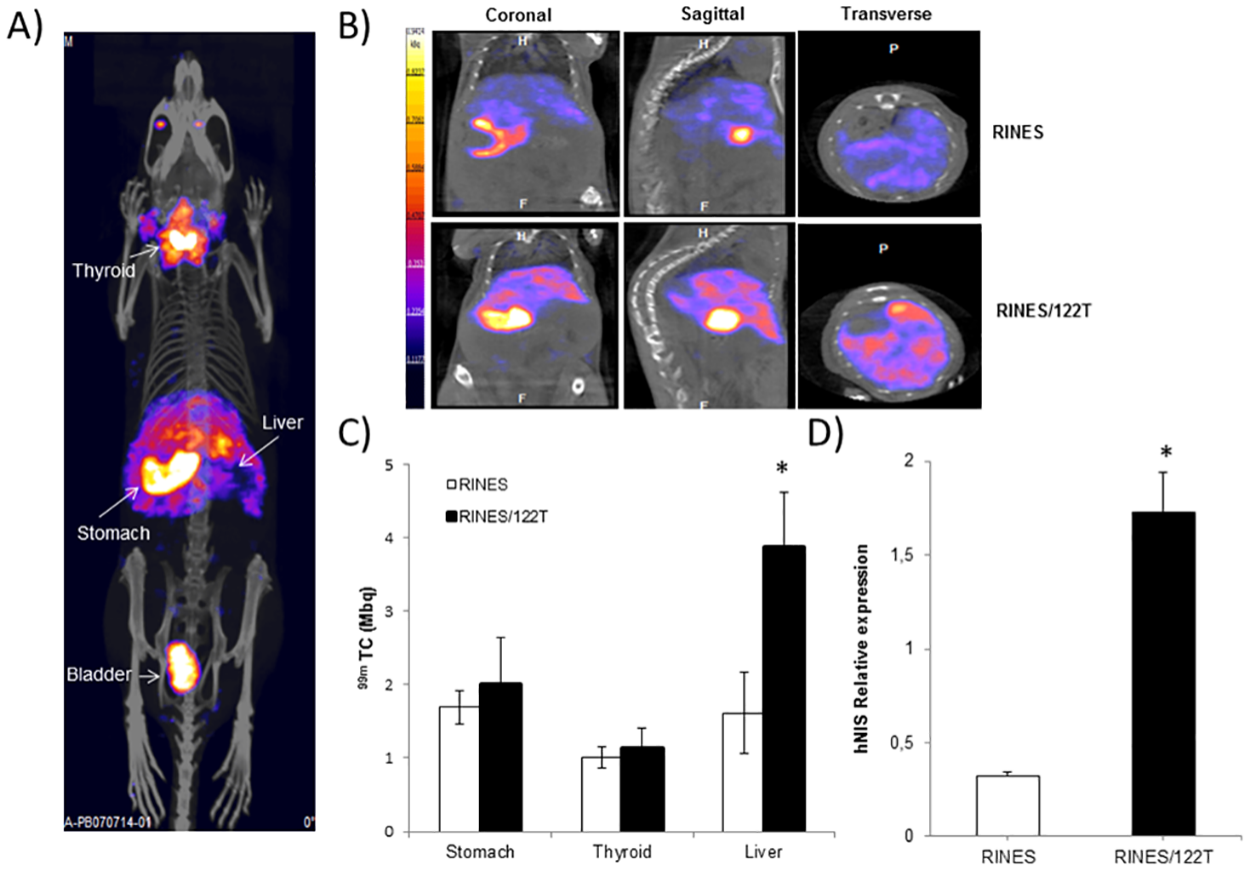
SPECT/CT imaging of miRNA-122 expression in the liver of mice. (A) A representative fused whole-body SPECT/CT image collected from one mouse injected hydrodynamically with the pRINES/122T plasmid. (B) Representative sagittal, coronal and transverse SPECT/CT images collected from the liver of one mouse from the pRINES and pRINES/122T group of mice. (C) Quantification of ^99m^Tc0_4_^-^ uptake in ROIs covering the liver of mice of the pRINES and pRINES/122T group of animals. (D). Quantitative RT-PCR analysis of hNIS transcript detected in the liver of 3 mice collected randomly from the pRINES and pRINES/122T group of animals. Error bars in C are the mean ± SEM (n = 5) of one representative experiment performed at least two times. Error bars in D are the mean ± SD (n = 3) of one representative experiment performed at least three times. Statistics by the two-tailed t-test, * P ≤ 0.05 compared with the pRINES control group.

We first attempted to monitor the expression of the liver specific miRNA 122 in animals. The RINES/122T and control pRINES plasmids were hydrodynamically injected in two groups of mice (n = 5 per group). A typical representative picture of fused SPECT and CT images is shown in Fig 5A. ^99m^TcO_4_^-^ tracer accumulated in the thyroid and the stomach as a result of endogenous expression of NIS, in the bladder as a result of ^99m^TcO_4_^-^ elimination in the urine, and in the liver as a result of gene transfer. Careful examination of all projections of the fused SPECT and CT sections demonstrated that signals were uniformly distributed inside the liver, confirming the great potential of hydrodynamic injection to deliver plasmid DNA efficiently to this organ. Fig 5B is an illustration of fused images collected from the coronal, sagittal, and transverse views of abdomen of mice from the pRINES/122T and control pRINES group. Regions of interest (ROIs) were drawn manually (liver, stomach) or automatically (thyroid) to quantify the radioactivity accumulated in the liver. The quantitative analysis of this experiment (Fig 5C) indicated that radiotracer accumulations in the stomach and the thyroid of mice were not significantly different (P ≥ 0.05) between the two groups of animals. In contrast, the mean value of radiotracer uptake (3.87 ± 0.15 MBq, n = 5) detected in the liver of the pRINES/122T group of mice was significantly different (P = 0.02) from the mean value detected in the control, pRINES group of mice (1.62 ± 0.15 MBq, n = 5). Finally to ascertain whether radiotracer uptake in the liver resulted in the expression of hNIS induced by miRNA-122, we performed a quantitative RT-PCR analysis on liver tissues using specific hNIS primers. Results (Fig 5D) indicated that the mean of relative expression of hNIS mRNA (1.73 ± 0.62) detected in the pRINES/122T group of mice was significantly higher (P ≤ 0.05) than that found in the pRINES group of mice (0.33 ± 0.22).

### Quantitative RT-PCR and SPECT/CT monitoring of miRNA expression in the tibialis skeletal muscle of mice undergoing muscular atrophy

The next experiments were dedicated to monitoring miRNA expression under physiopathological conditions. In our previous study [18], we demonstrated that the expression of miRNA-206 was significantly upregulated in the tibialis anterior muscle of mice undergoing muscular atrophy. Our objective here was to extend this work by examining whether other miRNA candidates may be also involved in the regeneration program of atrophied skeletal muscle tissues and to evaluate whether it might be possible to visualize their expression by SPECT/CT imaging. The well-known myomirs-133b and -1 were selected in addition to other non-specific muscle miRNAs such as miRNA-23a, -486, -221, previously alleged to be functionally involved in the biology of skeletal muscle cells [27].

**Fig 6:**
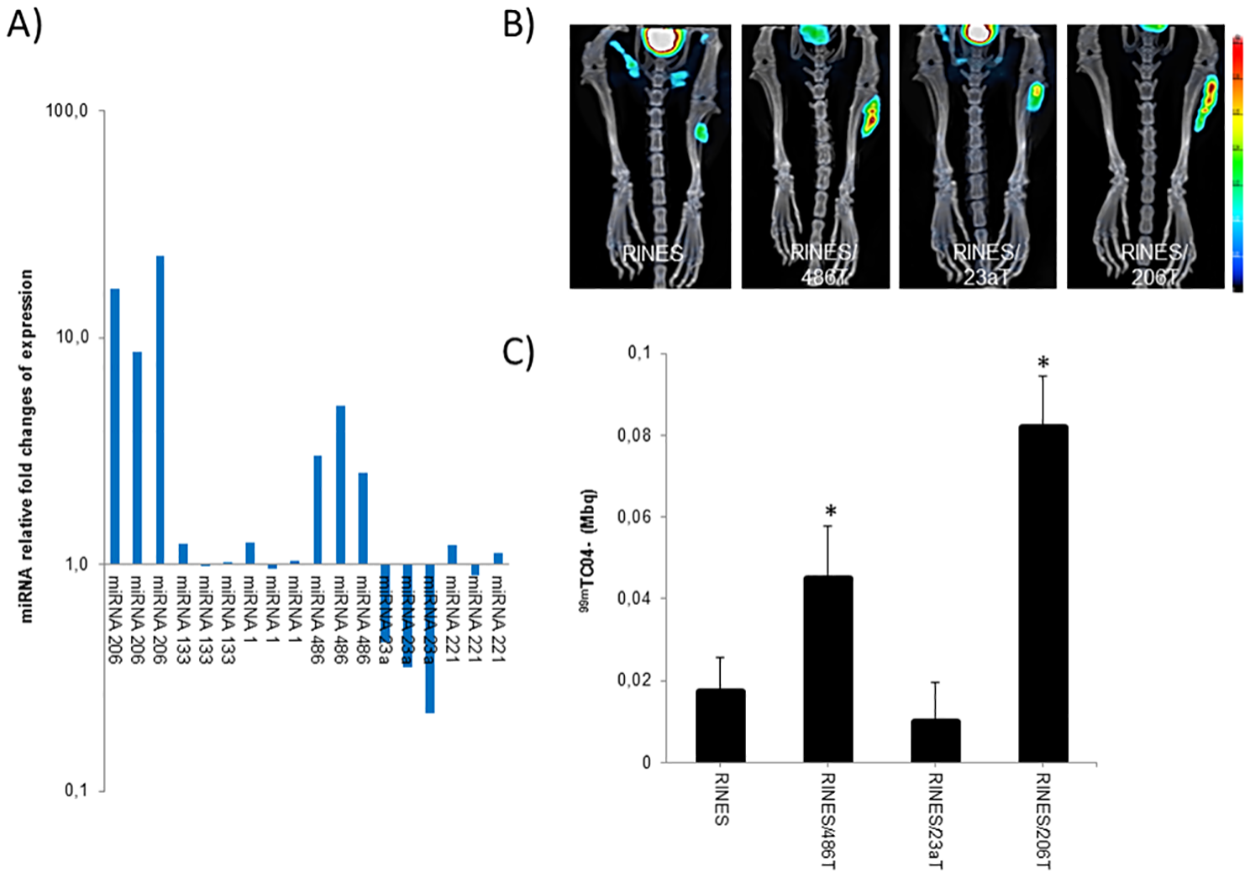
Quantitative RT-PCR analysis and SPECT/CT imaging of miRNA expression in tibialis skeletal muscle of mice undergoing muscular atrophy. A) Quantitative RT-PCR analysis of miRNA expression detected in the tibialis anterior muscles of mice collected 15 days after sciatic nerve transection to induce denervation and muscular atrophy. Results are expressed as miRNA relative fold change of expression relative to control, not denervated tissues, set arbitrarily to the value of 1. B) Typical fused SPECT/CT images collected from tibialis anterior muscles of one representative mouse in the pRINES/486T, pRINES/23aT, pRINES/206T and pRINES groups of animals. C). Quantification of ^99m^Tc0_4_^-^ uptake in ROIs covering the tibialis skeletal muscle of mice in the pRINES/486T, pRINES/23aT, pRINES/206T and pRINES groups of animals. Error bars in C are the mean ± SEM (n = 5) of one representative experiment performed three times. Statistics by the two-tailed t-test, * P ≤ 0.05 compared with the pRINES control group.

We first performed a screening procedure by real time PCR to identify the most deregulated miRNAs before visualizing their expression by SPECT/CT imaging. As shown (Fig 6A), amongst the 6 miRNAs screened, 15 days after sectioning the sciatic nerve of the mice, miRNA-206 was found to be the most significantly deregulated miRNA. The relative fold values of miRNA change of expression were 8.67, 16.40 and 22.83 for the 3 tissues examined. Surprisingly, we found that the relative miRNA expression of myomiR-1 (1.24, 0.95 and 1.04) and -133 (1.24, 0.98 and 1.22) was not significantly different (P = 0.25) from that quantified in control tissues. The same was observed for miRNA-221 as no significant difference (P = 0.22) was found between expression of this miRNA in atrophied tissues versus control, not-denervated tissues. In contrast, the expression of miRNA-23a was significantly down-regulated (P = 0.016) in the atrophied skeletal muscle tissues. Indeed, the relative fold values of miRNA change of expression were 0.45, 0.35 and 0.22 for the 3 tissues examined. On the contrary, the relative expression of miRNA-486 was significantly up-regulated (P = 0.042) in the denervated tibialis anterior muscle tissues. Indeed, the relative fold values of miRNA-486 change of expression were 3.02, 5.12 and 2.55 for the 3 mice examined.

Based on these data, we focused our experiment on miRNA-23a, -486 and -206 and managed to visualize their expression by SPECT/CT imaging. We administered the corresponding RINES plasmids intramuscularly and then sectioned the sciatic nerve of one leg of the mice before scanning the mice 15 days later. As control, we used the control pRINES plasmid, not regulated by miRNA. Typical examples of fused tomography and SPECT images collected from one mouse per group of animals are shown in Fig 6B. Results indicate that administration of the control pRINES and pRINES/23aT plasmids did not induce any substantial radiotracer accumulation in the left denervated tibialis anterior muscle of the mice. By contrast, administration of the pRINES/206T and pRINES/486T plasmids resulted in substantial radiotracer accumulation in the atrophied tibialis anterior muscle. This was confirmed by the quantitative analysis. The mean radiotracer values detected in the control RINES and RINES/23aT group of mice were low (0.017 ± 0.0085 MBq and 0.012 MBq ± 0.0095 respectively) and not significantly different (P = 0.34) from each other. In contrast, mean values of radiotracer uptake in the denervated tibialis anterior muscle of the RINES/486T and RINES/206T group of mice were higher (0.045 ± 0.012 MBq and 0.082 ± 0.014 MBq respectively) and significantly different to the control mean value (0.017 ± 0.0085 MBq). We finally performed an immunohistochemical analysis of hNIS expression between the pRINES and pRINES/206T group of mice. As expected and in line with the quantitative analysis, intense staining of hNIS protein was detected in the pRINES/206T tissues, as a consequence of induction of hNIS protein by miRNA-206, whereas no significant staining was detected in the control pRINES muscle tissues (S1 Fig).

### Real time monitoring of miRNA-23a expression during muscular atrophy development

The down-regulation of miRNA-23a in the tibialis anterior muscles of the mice in response to denervation was intriguing. First, because two recent publications report opposite results concerning the expression level of this miRNA in the skeletal muscle tissues of mice exposed to muscular atrophy [28, 29]. Second, because in other cellular contexts, miRNA-23a is reported to be an anti-apoptotic miRNA, overexpressed in several stress conditions that mediate its protecting function through the downregulation of APAF-1, a major constituent of the apoptosome machinery in cells [30–33]. A possible functional link of correlation between the expression of miRNA-23a and apoptosis of the denervated skeletal muscle tissues has not yet been reported. We thus attempted to investigate this point and decided to establish the exact kinetic of miRNA-23a expression during the development of atrophy. We performed this study by bioluminescence imaging using the RILES system that encodes for the luciferase reporter gene as this molecular imaging modality is less time- and money-consuming and was proved to be a reliable method to monitor miRNA expression in this pathological context [18].

**Fig 7:**
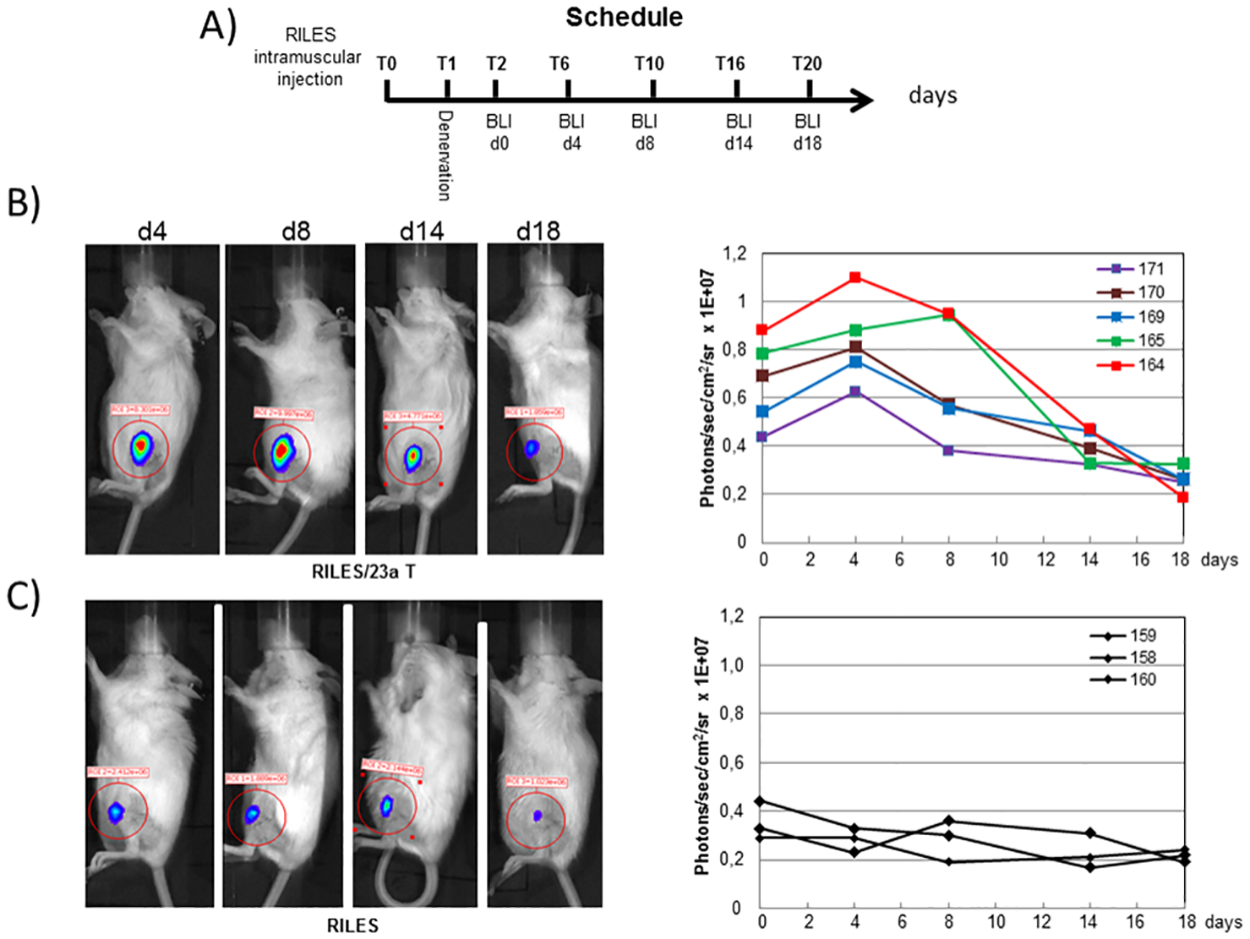
Non-invasive bioluminescence monitoring of miRNA-23a expression during development of skeletal muscle atrophy. A) Schematic representation of the procedure used to establish the kinetic of miRNA-23a expression in response to muscular atrophy. B). left panel, representative bioluminescence images collected from one representative mouse (n°125) from the pRILES/23aT group of animals at the indicated time points. Right panel, quantitative analysis of bioluminescence signals emitted from the pRILES/23aT group of animals and plotted as a function of time. C) Left panel, same as B, left panel, but for mouse n°159 from the RILES control group of animals. Right panel, same as B, right panel, but for the 3 mice from the RILES control group of animals.

The experimental approach used to establish the kinetic of miNRA-23a is shown in Fig 7A. Typical bioluminescence images collected at several time points from one mouse per group of animals are shown in the left panels of Fig 7B and 7C. To make apparent the kinetic of miRNA-23a expression, the quantitative bioluminescence values collected in each mouse from the pRILES and pRILES/23aT groups of animals were plotted as a function of time (right panels, Fig 7B and 7C). Results of this longitudinal analysis indicated that expression of miRNA-23a was high during the first phase of atrophy development (day 0 to day 5) for all the 5 mice investigated and started to decrease gradually from day 5 to day 15, becoming almost undetectable at day 20, the end point of our experiment. Interestingly, all the kinetic curves exhibit the same biphasic shape, similar to an inverted sigmoid curve, characterized by high and almost constant values at the early time points and low, almost negligible values at the end time points of the assay. In sharp contrast, kinetics of the RILES control group of mice were different, being similar to a straight line with constant and low bioluminescence values detected over time. The statistical analysis of the bioluminescence data indicated that the relative miRNA-23a expression values detected in the RILES/23aT group of mice (0.83 ± 0.18 x 10^7^) was almost 3 fold superior to the basal value detected in the RILES control group of mice (0.28 ± 0.05 x 10^7^). At day 20, there was no longer any significant difference (P = 0.45) between the RINES/23aT and the RINES control group of mice (0.26 ± 0.046 x 10^7^ versus 0.22 ± 0.032 x 10^7^).

### Western-blot analysis of APAF-1 and Caspase 9 expression in the atrophied tibialis anterior muscle tissues

**Fig 8:**
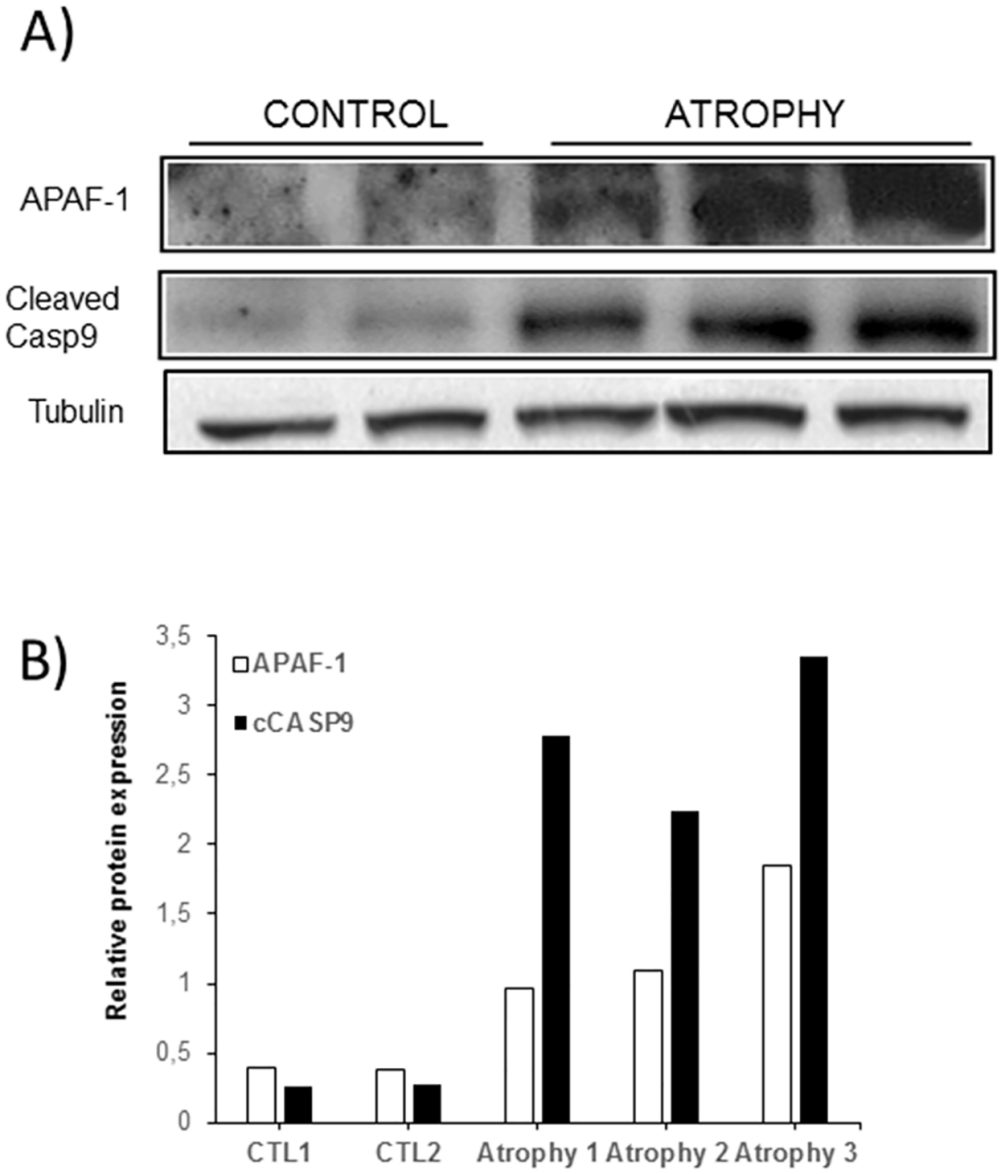
Western-blot analysis of APAF-1 and active form of caspase-9 expression in the atrophied skeletal muscle tissues. A) At autopsy of mice (day 20), the tibialis skeletal muscle tissues of 3 mice chosen randomly from the pRILES/23aT (atrophy) group of mice were collected and processed for a western-blot analysis using specific antibodies directed against APAF-1, Caspase 9 and tubulin. As control (control) we collected the tibialis skeletal muscle tissues of 2 naïve mice for which the sciatic nerve was not sectioned. Protein bands were revealed by chemiluminescence and autoradiography. B) Protein band intensities were quantified by densitometry using the image J software and expressed as relative protein expression by normalizing band intensities of APAF-1 and Caspase 9 to the band intensity of tubulin, used as loading control. This experiment was repeated at least twice with equivalent results.

To gain insight into the underlying biological consequence of miRNA-23a deregulation in response to muscular atrophy at late time points of atrophy development, we looked at the expression of APAF-1, as well as the main downstream target of the miRNA-23a/APAF-1 axis of regulation, Caspase 9. At autopsy, we collected the atrophied tibialis anterior muscle tissues of the RILES/23aT and RILES groups of mice and performed a western-blot analysis with specific APAF-1 and Caspase-9 antibodies (Fig 8A). The quantitative analysis of protein band intensities (Fig 8B) indicated that the relative mean of expression of APAF-1 proteins (2.24 ±0.87 x 10^4^) detected in atrophied tibialis anterior muscle tissues was higher and significantly different (P = 0.025) from the relative expression of APAF-1 proteins detected in control tissues (3.70 ± 0.70 x 10^3^). Interestingly, we also found that the upregulation of APAF-1 in the atrophied tissues correlated well with elevated expression of the active form of Caspase 9 in these same tissues. Indeed, the relative expression of the cleaved form of Caspase 9 protein was 4.69 x 10^4^ (± 0.94) in the atrophied tissues and 2.57 x 10^3^ (± 0.63) in the control tissues.

## Discussion

A current challenge in the field of miRNA is to monitor the expression pattern of miRNAs in a clinical context using compatible molecular imaging probes. Nuclear imaging reporter genes have a long history in the clinic [9] and remain one of the most sensitive probes for monitoring biological events in humans. In this context, the sodium iodide symporter is an attractive reporter gene [8] as it allows the accumulation of radioisotopes for both imaging and therapy. This theranostic property [9] has attracted interests in the field of gene therapy for image-guided radiation therapy that has been recently validated in humans [10–13].

In this study, we have demonstrated that positive monitoring of miRNA expression is feasible in a clinical setting using the RILES system coupled with hNIS as reporter gene, ^99m^Tc0_4_^-^ as radiotracer and a SPECT/CT camera. We show that radiotracer uptake in RINES-transfected cells reflects qualitatively and quantitatively the expression pattern of miRNA detected by quantitative RT-PCR and that the addition of sodium perchlorate, a competitive inhibitor of hNIS uptake, inhibits ^99m^Tc0_4_^-^ accumulation in cells. We monitored the endogenous expression of miRNA-122 in the liver of naive mice as well as other endogenously expressed miRNAs in the tibialis anterior muscle of a mouse model of muscular atrophy.

The advantage of our RINES molecular imaging system is that the expression of miRNAs signed by the emission of radionuclide signals in mice is more convenient than the miRNA-OFF monitoring system [5, 17]. The reduction or the absence of signals is constraining as it could reflect a dysfunction of the imaging scanner, inappropriate administration of the radiotracer, deregulation of the reporter genes and/or even cell death. Therefore, when extrapolated to the clinic, it could create ambiguity and doubt about the relevance of the clinical interpretation, which ultimately might false diagnosis as well as the decision to administer miRNA-based drugs. To generate an ON-signal, our RINES system employs an inducible genetic switch expression system that derived from a bacterial strain [26]. The long-term use of RINES in an immunocompetent individual may trigger an immune response against the bacterial origin of transcriptional regulator proteins as recently demonstrated with other prokaryotic regulatable gene expression systems [25, 34]. Nevertheless, this issue could be overcomed by humanizing the CymR repressor through a codon optimization strategy [35, 36]. Alternatively, this immune reactivity might be also viewed as an advantage to boost the immune response in a context of cancer gene immunotherapy. We also found in this study that in contrast to our previous results generated with the luciferase reporter gene [18], the use of hNIS as reporter gene did not provide a linear read-out in which radiotracer uptake in cells increased proportionally with the gradual ectopic expression of hNIS in cells. Saturation of radiotracer uptake in hNIS-transfected cells is seldom considered in preclinical studies performed with the hNIS as reporter gene. In most cases, a low level of hNIS expression is sufficient to enable radioisotope imaging of gene transfer *in vivo* as well as biodistribution of targeted cells administered in animals [14]. Few studies have addressed the quantitative aspect of the hNIS reporter gene, although defective accumulation of radiotracer uptake in cells expressing NIS was documented early on *in vivo* [7, 37–39]. An *in vitro* study by Vadysirisack et al. [20] evaluated the correlation between cell-surface hNIS protein levels and radioiodide uptake in cells expressing gradual amounts of exogenous hNIS. It was demonstrated that radioactivity uptake correlated with cell-surface NIS protein levels within a certain range of protein expression. Above this level, radioactivity accumulation in cells did not further increase but rather reached a plateau and saturated, as also found in our study. The mechanisms responsible for defective radiotracer uptake in cells expressing hNIS at high levels are still not totally understood and seem to come from several factors, the main ones being a defect in hNIS processing at the cell membrane, improper glycosylation/maturation of hNIS protein and change influx/efflux kinetics of ions in cells with high hNIS expression [7, 20, 40, 41]. Alternatively the use of another radionuclide such as ^125^I^-^ that do not rely on oxidation state to be active as for ^99m^TcO_4_^-^ [42, 43] might also view as a good alternative to improve the final outcome of the RINES. Several approaches have been evaluated to increase the rate of radiotracer uptake in hNIS expressing cells, including the treatment of cells with hormones [44, 45] and small molecules [46, 47]. The NIS of rat and mouse are also good candidates as these two orthologs of NIS are more efficiently targeted to the cell surface, providing a better outcome for radioisotope uptake [48, 49]. A codon-optimization procedure was also used to increase hNIS expression at the cell membrane. The optimized hNIS version improved ^125^I uptake by a factor of 2 in several cell types both *in vitro* and *in vivo*. This was correlated by a 4-fold increase in hNIS detected at the cell surface [50]. These studies warrant the use of murine NIS in mice for preclinical studies and the codon-optimized hNIS gene for potential clinical use.

We also established the dynamic expression pattern of miRNA-23a in response to muscular atrophy induced by sciatic nerve transection of one leg of the mice. Our data indicated that expression of this miRNA follows a biphasic process which corresponds to its level of expression at the early and late time points of atrophy development. This pattern of expression differs from the previously established expression pattern of miRNA-206 in response to muscular atrophy [18]. We provided evidence that expression of miRNA-206 is not constant during development of the disease but rather transient, characterized by strong inter-individual heterogeneity and variability in terms of longitudinal regulation. These results corroborate well with the now well-established role of miRNA-206 in the regeneration program of skeletal muscle undergoing atrophy. The miRNA-206 acts as a sensor miRNA, overexpressed in the damaged synaptic regions of muscle fibres of mice where it contributes to the regeneration of presynaptic regions through activation of the HDAC4 and FGFBP1 signalling pathways [51, 52]. Therefore it is not surprising that expression of this miRNA was subjected to great variability from mice to mice and also to great variation in temporal regulation. The regeneration of damaged neuromuscular junctions is indeed expected to be dependent on the severity of the injury, which *in fine* was not identical in each animal. Here, our primary aim was to monitor the dynamics of expression of other myomiRs in this pathological process. Our data indicated that out of the 6 miRNAs evaluated, only miRNA-206 and miRNA-23a were the most significantly deregulated. These results are also in agreement with a study demonstrating that changes in the expression pattern of miRNA in muscular atrophy are more qualitative than quantitative [53]. As the kinetic of miRNA-23a expression in response to muscular atrophy has not been previously reported, we decided to investigate this point by molecular imaging. The kinetics of miRNA-23a were almost similar for all mice, with a common shape, comparable to an inverted sigmoid curve, characterized by elevated expressions at early time points and a slow and progressive decrease of expression over time. At late time points, the expression of miRNA-23a was almost undetectable. Therefore, the observation that expression of these 2 miRNAs behaves differently in response to the same pathological stimuli and that expression of miRNA-23a did not seem to vary between mice, in contrast to miRNA-206, prompted us to suggest that regulation of miRNA-23a expression might be part of a more general process of regulation than the expression of miRNA-206.

To investigate this hypothesis, we focused on the late time point of atrophy development as the deregulation of miRNA-23a was more pronounced from day 5 to 18. It is recognized that the late stage of atrophy development, known as the adaptive response phase, is characterized by cell death through in part apoptosis [54, 55]. Furthermore, many reports have assigned a key biological role of miRNA-23a in the apoptotic program of many different cell types through modulation of APAF-1, a main compound of the apoptosome, that once bound to cytochrome C can promote the cleavage of the procaspase 9 and the production of the active form of this caspase[30–33]. We thus hypothesized that miRNA-23a might contribute to the apoptosis of atrophied muscle tissues through regulation of APAF-1. Our Western-Blot analysis performed at day 20 after denervation supports this statement. We found an opposite expression pattern between the loss of miRNA-23a and the upregulation of APAF-1 in atrophied mouse tissue that correlated well with detection of the active form of caspase 9. In contrast, in the non-denervated tissues the expression of APAF-1 and cleaved form of Caspase 9 proteins were almost undetectable. This again correlated well with the opposite expression pattern between APAF-1 and miRNA-23a. Indeed, expression of this miRNA was found to be high in these tissues by quantitative RT-PCR analysis (Fig 6A). These results suggest that the down-regulation of miRNA-23a in the late phase of muscular atrophy might be a detrimental event that could contribute to muscular atrophy development by promoting apoptosis through activation of Caspase 9. Notably, our assumptions are in agreement with a previous study [28] that demonstrated that ectopic expression of miRNA-23a reinforces the protection of skeletal muscle tissues from atrophy both *in vitro* and *in vivo* and that miRNA-23a transgenic mice show better resistance to skeletal atrophy induced by administration of dexamethasone. Mechanistically, miRNA-23a acts as a negative regulator of MAFbx/atrogen-1 and MuRF1 expression, two well-known ubiquitin ligases of the proteasome pathway responsible for the rapid proteolysis of skeletal muscle protein in the early stage of atrophy development [28]. Therefore, we propose that in addition to the role of miRNA-23a in the early stage of atrophy, the downregulation of miRNA-23a in the late development phase of this disease might also play a significant role by modulating the miRNA-23a/APAF-1/Caspase 9 axis of regulation. However additional studies are required to confirm this hypothesis and to define more precisely the exact mode of regulation exerted by miRNA-23a in this process.

## Conclusions

Our results demonstrated that engineering an inducible genetic switch expression system, such as RINES, is relevant to positively monitor the expression of miRNA in a clinical setting, using hNIS as reporter gene and a SPECT/CT camera. However, the immunogenicity of the prokaryotic transcriptional repressor used to switch-ON the RINES is currently a limitation to the possible application of the RINES to the clinic that might, nevertheless, be overcome by humanizing the CymR protein. We also underlined that the use of hNIS as reporter gene has some limitations, the main one being the saturation of radiotracer accumulation in cells when a high amount of hNIS protein is expressed at the plasma membrane. We established the kinetic of miRNA-23a expression in response to muscular atrophy. The information collected by bioluminescence imaging guided us to elucidate, at least partially, the biological significance of miRNA-23a deregulation in the apoptotic program of denervated tibialis skeletal muscle tissues. Our results suggest that the manipulation of miRNA-23a might have a dual therapeutic interest. Firstly, by controlling the expression of MAFbx/atrogin-1 and MurF1 in the early phase of atrophy development as demonstrated by others [28] and secondly by controlling the expression of APAF-1/Caspase 9 in the late phase of atrophy development as proposed in our study. Nevertheless, further investigations are required to fully validate the potential value of miRNA-23a as a therapeutic target to treat this chronic disease. Overall, our work warrants the use of the RINES system for imaging the expression of miRNA in preclinical animal models and its optimization for potential clinical use.

## Supporting information

S1 Fig Immunohistochemical detection of hNIS expression in the tibialis skeletal muscle tissues of mice injected intramuscularly with pRINES (left panel) or with pRINES/206T (right panel). One representative mouse per group from Fig 6B was sacrificed and the tibialis anterior muscle harvested and stained with a specific hNIS antibody. Pictures shown are representative of a staining performed from at least ten serial sectioned tissues. Scale bar: 200 μM.
(TIF)

S1 Table List of pRINES plasmids and primers sequences used in this study. (DOCX)
