# Supplementary figures and images for: Positive radionuclide imaging of miRNA expression using RILES and the human sodium iodide symporter as reporter gene is feasible and supports a protective role of miRNA-23a in response to muscular atrophy
Source: PLoS One. 2017 May 11;12(5):e0177492. doi: 10.1371/journal.pone.0177492 (PMC5426778; doi:10.1371/journal.pone.0177492)

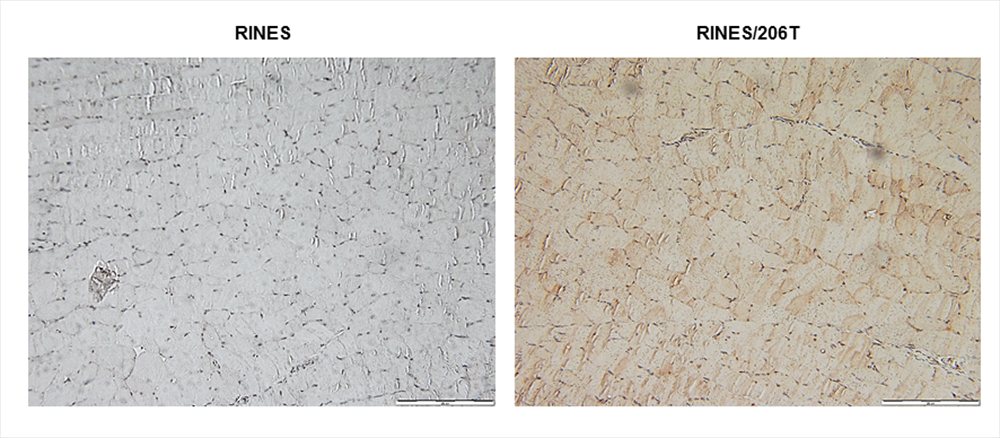

Supplement: S1 Fig — One representative mouse per group from Fig 6B was sacrificed and the tibialis anterior muscle harvested and stained with a specific hNIS antibody. Pictures shown are representative of a staining performed from at least ten serial sectioned tissues. Scale bar: 200 μM. (TIF) [file pone.0177492.s001.tif]
