# Supplementary material for: Positive radionuclide imaging of miRNA expression using RILES and the human sodium iodide symporter as reporter gene is feasible and supports a protective role of miRNA-23a in response to muscular atrophy
Source: PLoS One. 2017 May 11;12(5):e0177492. doi: 10.1371/journal.pone.0177492 (PMC5426778; doi:10.1371/journal.pone.0177492)
Supplement: S1 Table — (DOCX) [file pone.0177492.s002.docx]

**SUPPLEMENTARY DATA**

**List of RINES plasmids used.**

| **RINES plasmids** | **RNAi Targeting sequence subcloned into 3’-untranslated region of CymR transcript** |  |
| --- | --- | --- |
| pRINES | No RNAi targeting sequence |  |
| pRINES/122T | | GGCCGCAA**CAAACACCATTGTCACACTCCA**TAGTA**CAAACACCATTGTCACACTCCA**CGAT**CAAACACCATTGTCACACTCCA**ATGC**CAAACACCATTGTCACACTCCA**GCTAGCTTA |
| pRINES/23aT | GGCCGCAA**GGAAATCCCTGGCAATGTGAT**TAGTA**GGAAATCCCTGGCAATGTGAT**CGAT**GGAAATCCCTGGCAATGTGAT**ATGC**GGAAATCCCTGGCAATGTGAT**GCTAGCTTA |  |
| pRILES/486T | GGCCGCAA**CTCGGGGCAGCTCAGTAGAGGA**TAGTA**CTCGGGGCAGCTCAGTAGAGGA**CGAT**CTCGGGGCAGCTCAGTAGAGGA**ATGC**CTCGGGGCAGCTCAGTAGAGGA**GCTAGCTTA |  |
| pRILES/206T | GGCCGCAA**CCACACACTTCCTTACATTCCA**TAGTA**CCACACACTTCCTTACATTCCA**CGAT**CCACACACTTCCTTACATTCCA**ATGC**CCACACACTTCCTTACATTCCA**GCTAGCTTA |  |

Bold and underlined : the four-block sequence complementary to miRNAs of interest.

**List of primers used:**

For mRNA analysis

hNIS Forward: 5’-ACCTGACAATGGAGGCTCTC-3’

hNIS Reverse: 5’-TCTGTCTTCACGTGGCATCT-3’

6S Forward: 5’-CCAAGCTTATTCAGCGTCTTGTTACTCC-3’

6S Reverse: 5’-CCCTCGAGTCCTTCATTCTCTTGGC-3’

For miRNA analysis

miRNA-1: 5’-CCGGTGGAATGTAAAGAAGTATGTAT-3’

miRNA-133a: 5’-GTCCCCTTCAACCAGCTGAA-3’

miRNA-206: 5’-TGGAATGTAAGGAAGTGTGTGG-3’

miRNA-486: 5’-GTACTGAGCTGCCCCGAG-3’

miRNA-23a: 5’-CTACATTGTCTGCTGGGTTTC-3’

miRNA-221: 5’-CTACATTGTCTGCTGGGTTTC-3’

U6: 5’-CGCAAGGATGACACGCAAATTC-3’
